# Time series analysis of malaria in Afghanistan: using ARIMA models to predict future trends in incidence

**DOI:** 10.1186/s12936-016-1602-1

**Published:** 2016-11-22

**Authors:** Mohammad Y. Anwar, Joseph A. Lewnard, Sunil Parikh, Virginia E. Pitzer

**Affiliations:** 1 Department of International Health, Johns Hopkins Bloomberg School of Public Health, Baltimore, MD USA; 2 Department of Epidemiology of Microbial Diseases, Yale School of Public Health, New Haven, CT USA

**Keywords:** Malaria, Prediction, Afghanistan, Environment, Autoregressive model

## Abstract

**Background:**

Malaria remains endemic in Afghanistan. National control and prevention strategies would be greatly enhanced through a better ability to forecast future trends in disease incidence. It is, therefore, of interest to develop a predictive tool for malaria patterns based on the current passive and affordable surveillance system in this resource-limited region.

**Methods:**

This study employs data from Ministry of Public Health monthly reports from January 2005 to September 2015. Malaria incidence in Afghanistan was forecasted using autoregressive integrated moving average (ARIMA) models in order to build a predictive tool for malaria surveillance. Environmental and climate data were incorporated to assess whether they improve predictive power of models.

**Results:**

Two models were identified, each appropriate for different time horizons. For near-term forecasts, malaria incidence can be predicted based on the number of cases in the four previous months and 12 months prior (Model 1); for longer-term prediction, malaria incidence can be predicted using the rates 1 and 12 months prior (Model 2). Next, climate and environmental variables were incorporated to assess whether the predictive power of proposed models could be improved. Enhanced vegetation index was found to have increased the predictive accuracy of longer-term forecasts.

**Conclusion:**

Results indicate ARIMA models can be applied to forecast malaria patterns in Afghanistan, complementing current surveillance systems. The models provide a means to better understand malaria dynamics in a resource-limited context with minimal data input, yielding forecasts that can be used for public health planning at the national level.

**Electronic supplementary material:**

The online version of this article (doi:10.1186/s12936-016-1602-1) contains supplementary material, which is available to authorized users.

## Background

Afghanistan is a landlocked country located at the crossroads of several geographical regions [1]. Although generally arid, there are numerous rain- and snow-fed rivers [2], where historically human settlements formed at their surroundings, providing fertile ground for mosquito-borne diseases such as malaria. Major malaria vectors in the country are *Anopheles stephensi, Anopheles superpictus, Anopheles hyrcanus, Anopheles pulcherrimus, Anopheles culicifacies,* and *Anopheles fluviatilis* [3, 4]. The major species are *Plasmodium vivax* (70–95%), followed by *Plasmodium falciparum* [5, 6]. Malaria is endemic and seasonal in Afghanistan and the surrounding region [7, 8]. Although varying figures are given for the number of people at risk for malaria [5, 9, 10] the consensus is that significant numbers reside in malaria-endemic regions, notably in the semiarid eastern provinces, rice growing northern provinces, and greener areas under 1500 m in elevation [11]. In recent times, there have also been outbreaks of malaria in non-traditional highland provinces above 2000 m, where malaria transmission was previously not believed to occur (e.g. Bamiyan province in the year 2000, with elevation of over 2400 m) [12].

A particular problem with understanding the dynamics of malaria in Afghanistan is the scarcity of consistent and systemic information sources due to a combination of lack of infrastructure and constant civil unrest. In this unstable setting, not much is known about the intensity, magnitude, and temporal dependence of epidemic patterns over time. Only recently has a systemic surveillance system been put in place [13], but the scope is limited and mostly confined to accessible regions. Reporting is based on passive case finding from facilities by health professionals. It is retrospective and often late to detect emerging patterns. Hence, a tool to actively predict future trends is needed, especially one with the capability of producing good results in a resource-poor and war-torn setting like Afghanistan.

The increasing availability of data on climatic, geographic, and environmental determinants of transmission encourages consideration of these factors together with clinical data to prepare early warning signals of changing malaria trends in modern public health surveillance [6]. It has been proposed that variables like air temperature [14], rainfall [15], altitude [16], humidity [17], vegetation index [18], and even surface water fraction [19] increase predictive power of malaria models [20], not only for short periods, but also over longer timescales [21]. Tools used to measure the association between these factors and malaria patterns have included linear regression [22], Poisson regression [23], Spearman’s correlation [24], non-linear methods [25], and autoregressive time series methods [26].

In this paper, an autoregressive integrated moving average (ARIMA) model was used, applied to time series data of malaria incidence in Afghanistan. The model looks for temporal dependence between successive observations [27]. Due to the transmissibility and seasonality of malaria, models with an ARIMA structure have more predictive power compared to other methods [28]; such models have been applied to predict numerous infectious diseases with similar periodic patterns over the past decades [29, 30]. Another advantage of the ARIMA approach is the relative simplicity and stability of the model in predicting malaria cases in a context where political unrest and poor resources lead to a lack of detailed data, which makes it difficult to calculate parameters needed for construction of more complex models of malaria [31]. Remotely-sensed climate and environmental data were incorporated to test associations with climate and improve the predictive power of proposed model [32].

## Methods

### Malaria data

Models forecasting monthly malaria incidence throughout Afghanistan were developed. Data were available from cases reported nationwide across all regions of Afghanistan over the period from January 2005 to September 2015 through Health Management Information System (HMIS), a Ministry of Public Health-operated database [33], which collects reports from public health facilities accessed by over 85% of the population [34]. These reports capture passively detected cases from the public health system, and include both parasitologically confirmed and clinically suspected cases referred to outpatient departments. Inclusion of clinically suspected cases as numerator makes results prone to overestimation, but after accounting for significant underreporting of confirmed cases due to the lack of laboratory facilities, and the fact that around 15% of the population still lack access to health services and could have higher incidences compared to those under coverage, the numbers approximate those reported by the World Health Organization (WHO) for Afghanistan (the only available reference) [5].

No public census has been conducted in Afghanistan since 1979 [35], and other sources of demographic data [e.g. WHO, International Monetary Fund (IMF), Central Statistics Office (CSO)] cannot be corroborated with each other. In addition, utilization of health services was not homogenous throughout the study period (Fig. 2c), as the number facilities has risen from under 1000 to over 2000 centres since 2004. Hence, data on the total monthly new outpatient department visits were used as denominator in order to control for demographic and reporting trends. To verify that this did not lead to a bias in the trends over time due to recent changes in outpatient health service utilization occurring primarily in regions of either low or high malaria incidence, the overall of trend of malaria obtained after adjustment was compared with the weighted average of individual trends of provinces adjusted for their level of health service utilization.

### Climate/environmental data

Satellite-based measures of meteorological and environmental variables used to aid forecasting were available from the earth observing system data and information system (EOSDIS). Precipitation (mm/month), surface relative humidity (daily data, averaged by month), enhanced vegetation index (EVI) [36] (monthly average land greenness fraction), and surface air temperature (daily data, averaged by month) were assessed for Afghanistan as potential predictors. Both Malaria and climate data were provided as Additional files 1, 2, 3, 4 and 5.

### Statistical procedure

ARIMA models were developed to forecast malaria incidence based on temporal autocorrelation present in the incidence data. The dataset was split into a training period (January 2005 to December 2013), used as a platform for creating the ARIMA models, and a validation period (January 2014 to September 2015), which was used to test the models’ predictive ability.

ARIMA models provide *n*-step–ahead predictions based on patterns of temporal dependence in time series data. The notation (*p*,*d*,*q*) × (*P*,*D*,*Q*)_*S*_ describes the composition of temporal patterns considered for forecasting: these include autocorrelation over a maximum of *p* months or over *P* periods, each of length *S* = 12 months in our dataset; differencing over *d* adjacent months or *D* periods; and moving averages sustained over *q* months or *Q* periods. To determine patterns best describing the malaria time series, we followed the Box-Jenkins approach to ARIMA model selection, consisting of three steps [37]. First, malaria incidence was plotted against time to detect and correct for non-stationarity of the time series (Fig. 2), and identified autoregressive and moving average terms needed by calculating the autocorrelation (ACF) and partial autocorrelation (PACF) functions. Next, models of varying orders were fitted, and compared via the Akaike information criterion (AIC) [38] to assess improvements in fit while penalizing model complexity. Last, temporal autocorrelation was confirmed to have been no longer present in model residuals using the Ljung-Box test [39].

The selected models were used to generate forecasts for the validation period from January 2014 to September 2015 as 1-, 2-, 3-, 6-, and 12-month ahead forecasts. The rationale was to find which model works better for real-time, short-term surveillance objectives as compared to longer-term (up to yearly) prediction of future malaria patterns.

Out-of-sample forecast accuracy across models was compared by calculating the mean square error (MSE) and the predictive *R* ^2^, which is equal to 1 – (mean squared error)/(variance of the time series). Similar to the coefficient of determination, predictive *R* ^2^ tends toward one as models explain more observed heterogeneity in a time series, but can also take on values less than zero when the mean of the time series would provide a better estimate than model-based forecasts. Lastly, model forecasts, along with 95% prediction intervals, were plotted and compared against the observed data between January 2014 and September 2015.

It was evaluated whether incorporating meteorological and environmental variables improved the models’ fit and forecasting ability. Predictors were selected using a standard “pre-whitening” approach to identify whether each variable and the malaria time series were associated after adjusting for shared patterns of temporal dependence [40]. ARIMA models were selected and fitted to each climatic predictor, then fitted ARIMA models of the same order to the malaria time series. The cross-correlation function was evaluated between residuals series from the two models to identify lags at which anomalies in the climate variables explained unaccounted-for heterogeneity in malaria incidence. Lags found to be significantly correlated with malaria residuals were incorporated into the base ARIMA model as external regressors. Models with external regressors were used for both short- and long-term predictions; regressors were forecasted with the corresponding number of time steps before being incorporated into the malaria prediction models whenever predictive horizons exceeded the available data on these variables.

R statistical package (R Core Development Team, Vienna) and Stata v12 (StataCorp, College Station, TX) were used to carry out the analyses.

## Results

**Fig. 1:**
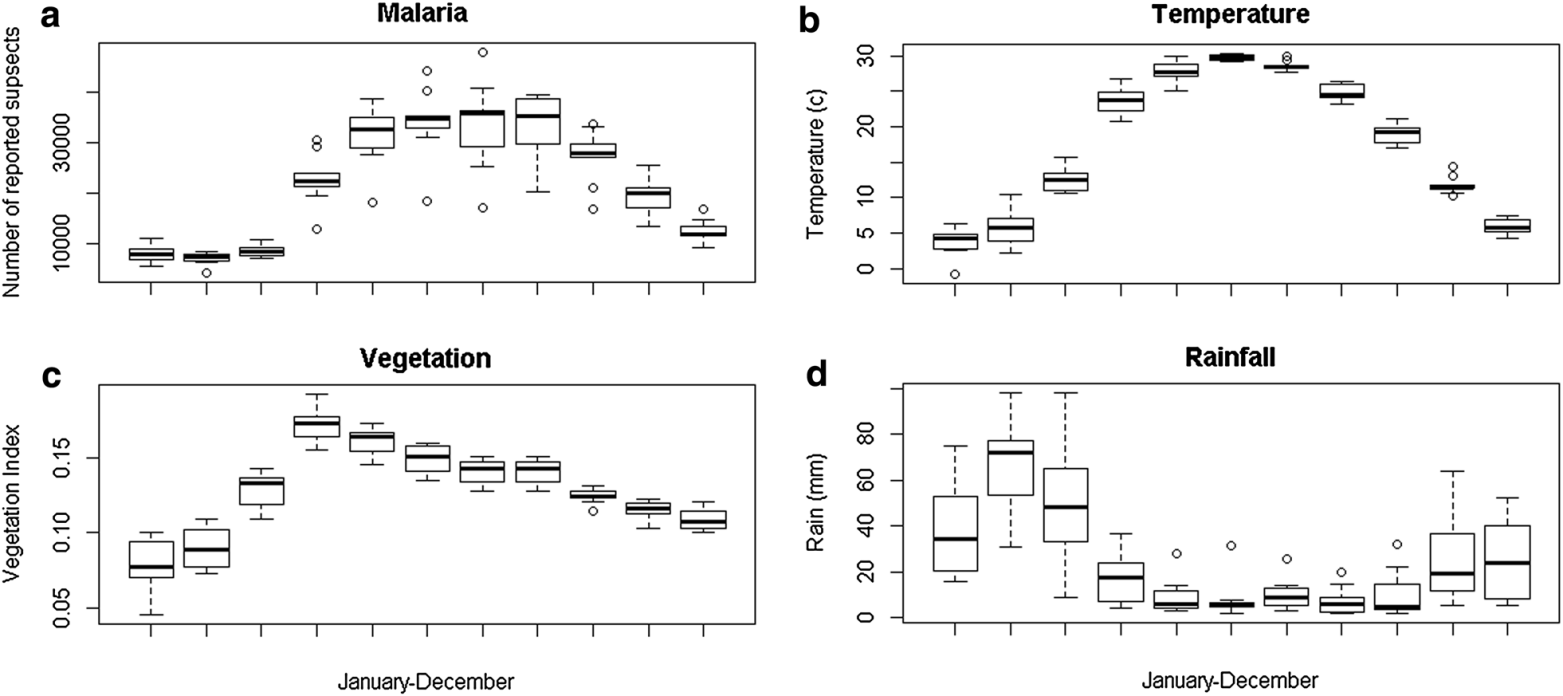
Seasonal variation of malaria and environmental variables (2005–2014). From *top left* in clock wise order: **a** monthly variation of malaria, **b** monthly variation of Temperature, **d** monthly variation of rainfall, **c** monthly variation of vegetation index

The dataset covers 129 months, starting from January 2005 to September 2015. The total number of suspected (including confirmed) malaria cases reported throughout the period was 2,243,452 with a mean of 20,772 clinical cases per month, and standard error of 1097 cases. The number of reported cases per month ranged from 4309 to 47,779, consistent with the seasonal nature of malaria in the country. Indeed, looking at the seasonal distribution of cases over the years (Fig. 1a), malaria cases peak between June–September, around the time when temperature is high and rainfall low (Fig. 1b, d), and lag vegetation variation by few months (Fig. 1c). Geographically, in descending order, eastern (1,351,530), north eastern (366,635), northern (239,230), southern (145,220), central (87,227), and western (53,610) regions report the most cases.

**Fig. 2:**
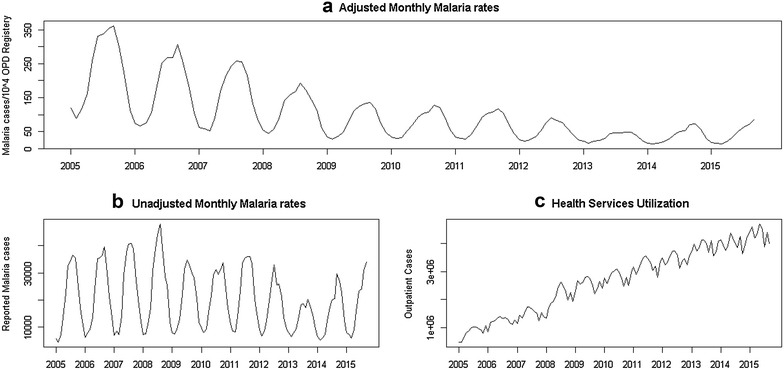
Malaria cases per month, from January 2005 up to September 2015, reported from health facilities throughout Afghanistan. **a** Adjusted for monthly cases per 10,000 outpatient clients, as reported from health facilities. **b** Unadjusted monthly malaria cases. **c** Total number of outpatient cases, reflecting trends health services utilization and reporting. Although the unadjusted data do not exhibit any trend beyond seasonality, because fewer centers were reporting at the beginning of the period (around 1000 centers compared to well over 2000 in 2015 [42]) and health services utilization increased substantially and proportionally for all parts of the country, adjustment was necessary to account for under-reporting. Subsequent analyses were performed using the adjusted rates

Malaria notifications have proportionally declined relative to the total number of outpatient visits consistently since the beginning of 2005, with seasonal pattern of 12-month in length, which has decreased in amplitude over time (Fig. 2a). The overall (linear) trend in malaria cases per 1000 outpatient visits was −27 (CI −34, −21) per year, compared with a population-weighted mean of −32 (CI −47, −18) cases per 1000 outpatient visits per year for provinces individually; thus the rate of decline was statistically the same for provinces as for the country as whole.

**Fig. 3:**
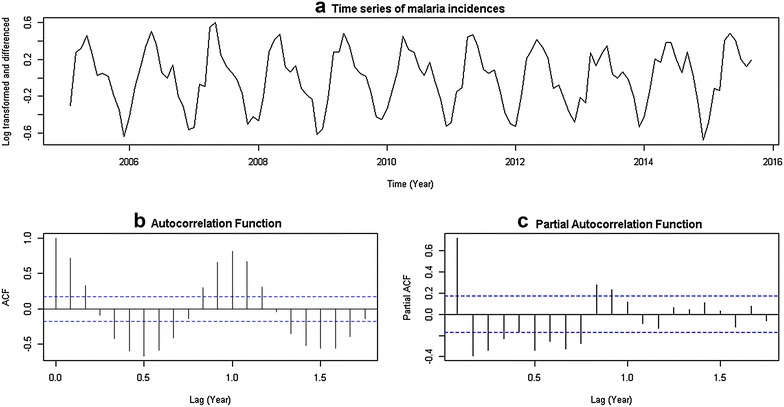
**a** Log-transformed and differenced malaria incidence (monthly incidence/all outpatients) over time, from January 2005 to September 2015. **b** Autocorrelation (ACF) and **c** Partial autocorrelation function (PACF) of malaria time series data

The time series data were log-transformed then differenced to stabilize the variance and remove the linear trend, respectively (Fig. 3a). The resulting time series exhibits a faint, statistically non-significant second periodic peak after the first, possibly due to distinct *P. vivax* and *P. falciparum* cycles [41]. Based on the ACF and PACF patterns (Fig. 3b, c), an ARIMA model of order (4,1,1) × (1,0,1)_12_, (Model 1, AIC = −145.02) was selected and fitted (with the consideration of first degree differencing). The residuals did not show a statistically significant autocorrelation pattern (Ljung-Box test *p* = 0.4067) (Additional file 6: Annex 1; Table 1). For comparison, a more parsimonious ARIMA model of order (1,1,1) × (1,0,1)_12_ (Model 2, AIC = −132.18) was also considered; however, a marginal degree of temporal autocorrelation persisted in the residuals of Model 2 (*p* = 0.052) (Additional file 6: Annex 1).

**Table 1:** Coefficients and standard errors of parameters of both ARIMA models

	Coefficient	Standard error
Coefficients, and standard errors of the parameters of the ARIMA Model 1 [(4,1,1) × (1,0,1)_12_]		
Intercept	−0.0197	0.0186
Non-seasonal AR(1)	0.6745	0.1342
Non-seasonal AR(2)	0.0120	0.1162
Non-seasonal AR(3)	−0.0068	0.1161
Non-seasonal AR(4)	−0.3169	0.0991
Non-seasonal MA(1)	−0.7784	0.0890
Seasonal AR(1)	0.9998	0.0016
Seasonal MA(1)	−0.9576	0.1424
Coefficients, and standard errors of the parameters of the ARIMA Model 2 [(1,1,1) × (1,0,1)_12_]		
Intercept	−0.0209	0.0840
Non-seasonal AR(1)	−0.0035	0.0028
Non-seasonal MA(1)	−0.0524	0.0163
Seasonal AR(1)	0.9996	0.0000
Seasonal MA(1)	−0.9154	0.0094
Coefficients, and standard errors of the parameters of the ARIMA Model 2 [(1,1,1) × (1,0,1)_12_]—Lag2 EVI		
Intercept	−0.0408	0.0953
Non-seasonal AR(1)	0.7777	0.0729
Non-seasonal MA(1)	−1.0000	0.0033
Seasonal AR(1)	0.9993	0.0023
Seasonal MA(1)	−0.8782	0.1833

Both models were used to compare the observed versus predicted malaria incidence from January 2014 to September 2015. For one-step ahead predictions, the estimated values show less dispersion using Model 1 compared to Model 2 (reduction in MSE of 10%) (Table 2); this suggests Model 1 may be better suited for short-term, out-of-sample malaria forecasting. For longer-term prediction, the MSE and predictive *R* ^2^ of both models were compared. The values estimated for 2- , 3- , 6- and 12-step ahead approaches exhibit generally better predictive power for Model 2 at longer time steps, despite its poorer within-sample fit as measured by AIC (Table 3).

**Table 2:** Comparison of 1-step ahead models with and without external regressors

ARIMA model	Lag-2 EVI coefficient	Standard error	AIC	MSE
Model 1			−145.02	0.0088
Model 1 with Lag-2 EVI	0.6287	0.4719	−121.99	0.0142
Model 2			−132.18	0.0098
Model 2 with Lag-2 EVI	0.1728	0.7304	−147.92	0.0086

**Table 3:** Model forecasting and validation for 2-, 3-, 6-, and 12-step ahead predictions for both models, with or without the external regressor (EVI at a lag of 2 months) over the period from January 2014 to September 2015

Steps ahead	Model							
	Model 1				Model 2			
	(4,1,1) × (1,0,1)12		(4,1,1) × (1,0,1)12—lag 2 vegetation		(1,1,1) × (1,0,1)12		(1,1,1) × (1,0,1)12—lag 2 vegetation	
	MSE	R2	MSE	R2	MSE	R2	MSE	R2
2	0.041	0.897	0.045	0.887	0.045	0.887	0.041	0.897
3	0.075	0.812	0.075	0.810	0.064	0.838	0.066	0.833
6	0.113	0.634	0.132	0.666	0.107	0.729	0.107	0.729
12	0.160	0.595	0.168	0.575	0.145	0.633	0.144	0.638

Subsequently it was assessed whether incorporating external climate regressors improved the predictive power of proposed models. The correlation coefficients between the covariate data and the residuals of the ARIMA model fit to the time series over a range of lags are presented in Additional file 7: Annex 2. Using the pre-whitening approach, it was found that only EVI with a lag of 2 months was significantly correlated with the malaria outcome (pairwise correlation = 0.2012, *p* = 0.0318) (Additional file 7: Annex 2). After fitting Models 1 and 2 with EVI as an external regressor, we found the simpler model (Model 2) demonstrated improved within-sample model fit (AIC = −147.69), whereas fit for Model 1 was not improved (AIC = −121.99) (Table 2). Incorporating EVI marginally improved the accuracy of one-month ahead forecasts from Model 2 (Table 2). Even though the forecasted vegetation index itself was not a significant predictor, adjusting for EVI in Model 2 affected the estimates of the other contributing parameters, in particular strengthening the non-seasonal autoregressive and moving average terms (Table 1), leading to a better overall model fit. As found in the earlier analysis, Model 2 had generally better longer-term predictive power compared to Model 1, and accounting for lag-2 EVI further improved the predictive power by a small factor (Table 3).

**Fig. 4:**
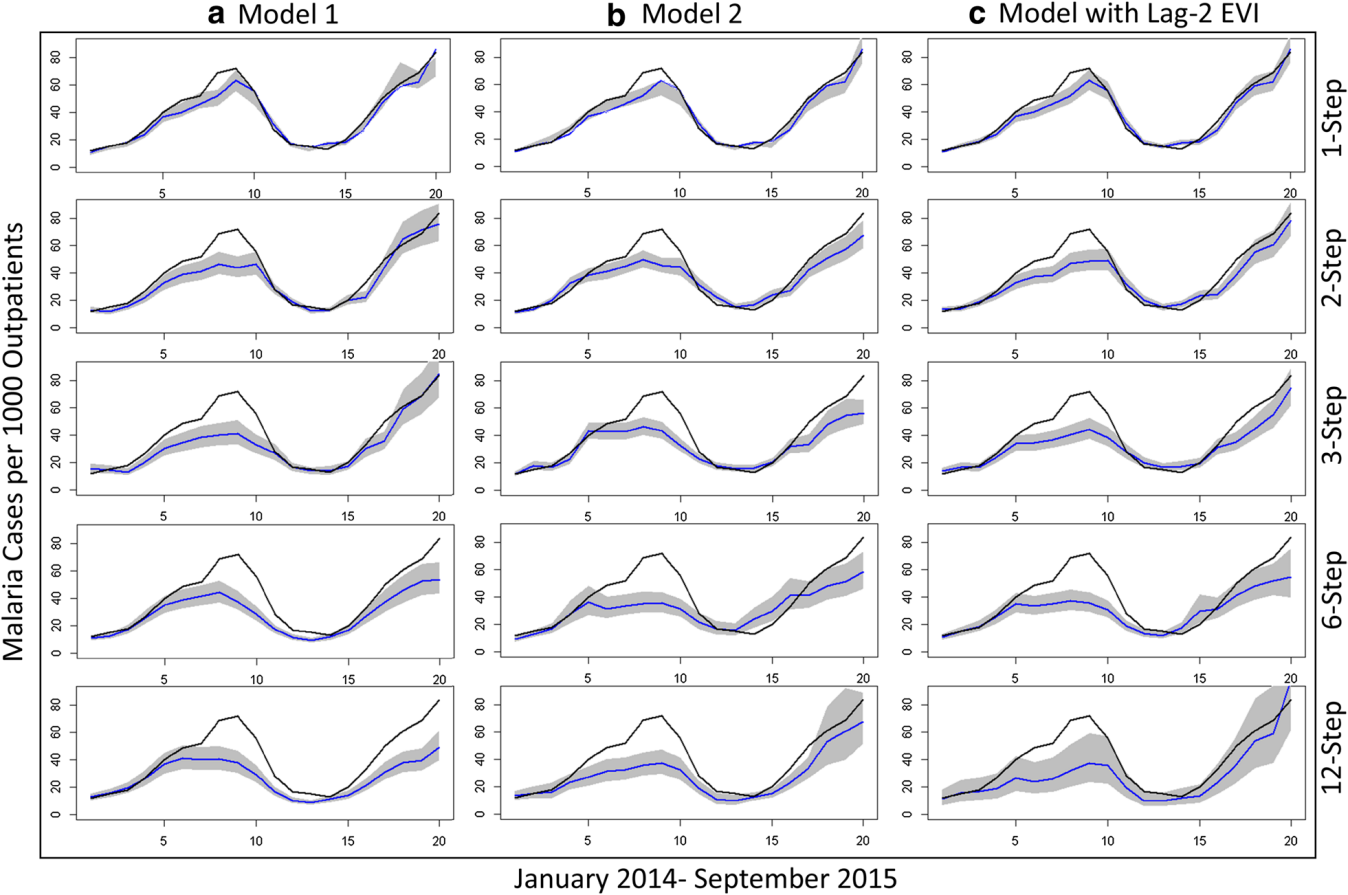
Out-of-sample prediction of different models. Columns (*Left* to *right*): **a** Model 1, **b** Model 2, and **c** Model 2 with enhanced vegetation index (EVI) at a lag of 2 months. The *rows* (from *top* to *bottom*) show 1-, 2-, 3-, 6-, and 12-ahead predictions. The *black lines* represent the observed adjusted time series data, while the *blue lines* represent the predicted values and the *grey regions* correspond to 95% prediction intervals

Figure 4 demonstrates the 2-, 3-, 6-, and 12-step ahead predictions and fitted values for the multiplicative ARIMA (4,1,1) × (1,0,1)_12_ model (Model 1), (1,1,1) × (1,0,1)_12_ model (Model 2), and Model 2 with lag-2 EVI. Model forecasts for the expected number of clinically suspected malaria cases up to December 2016 are presented in Additional file 8: Annex 3, using 12-step ahead predictions from Model 2; these estimates depend on the assumptions highlighted in Additional file 8: Annex 3.

## Discussion

While the overall number of malaria cases reported to the Health Management Information System in Afghanistan has remained fairly constant, analysis indicates malaria incidence and the intensity of seasonal epidemics as a proportion of the total number of outpatient clients have been steadily declining (by greater than 75%) since 2005 [5]. This perhaps can be attributed to recent efforts to expand health services in the country [34], which may have resulted in a general drop in communicable diseases, including malaria [43]. Furthermore, wider implementation of preventive measures such as insecticide-treated nets in recent years, even in remote and impoverished regions [44], have been shown to have a negative correlation with malaria incidence [45]. In addition, substantial increase in number of trained health worker in recent years helped maximize the effect of malaria control programmes [46]. It might be even possible to credit these designed intervention as the major determinant of malaria trend in the country.

After adjusting for these trends in malaria incidence, two ARIMA models were evaluated. The best fit to the data was obtained with a (4, 1, 1) × (1, 0, 1)_12_ model. Thus, the number of malaria cases in a given month can be estimated based on the number of cases occurring 1, 2, 3, 4, and twelve months before, after adjustment for negative seasonal and non-seasonal moving averages (i.e. a slight decrease in average cases in a given month compared to the prior month and the same month but in the previous year, respectively). Although this model is a good fit for short-term 1-step ahead prediction, it does not perform as well for longer-term predictions.

The second model, which is a (1, 1, 1) × (1, 0, 1)_12_ model, indicates that the number of malaria cases can be estimated from cases occurring one month and 12 months before. Again, the moving average parameters indicate a drop in magnitude of average cases in a given month compared to 1 and 12 months before. Although this model does not provide as good a fit to the observed data as the model above, it nonetheless has better long-term predictive power, and estimated averages remain close to the observed data. Furthermore, the fit and predictive power of the second model can be improved with the addition of environmental variables.

Several climate and environmental variables have been associated with malaria incidence [14, 20]. To measure associations between these variables and malaria incidence in Afghanistan, the data were pre-whitened to facilitate the evaluation of possible correlation between two time series after accounting for temporal and seasonal autocorrelation. In the absence of pre-whitening, significant correlations existed between malaria and average monthly rainfall (0–3 month lags), vegetation (0–3 month lags), and temperature (0–3 month lags) (Additional file 7: Annex 2), which are likely attributable to common seasonal patterns. After pre-whitening, it was found that only EVI had a significant association with malaria at a lag of 2 months. Thus, average malaria cases might depend on how green the environment was (i.e. the amount of vegetation covering the environment, as measured by EVI) 2 months before.

Incorporating EVI as an external regressor at a lag of 2 months improved the predictive power of Model 2, especially for 2-, 6- and 12-steps ahead predictions; the same did not happen with Model 1. Although the improvement is not substantial, it is nonetheless helpful to empower surveillance bodies in the country to sharpen their predictions, and to understand how much of a role environment plays in malaria dynamics in the country.

The finding that vegetation is correlated with malaria cases in Afghanistan is in line with other studies using remote sensing data in close or distant regions that found such association with lags between (0–3) months [18, 47]. Although strong evidence exists for an effect of temperature and rainfall on malaria, results did not point to any statistically significant correlations with these variables after controlling for the seasonal and autoregressive patterns. The reason might be our assumption that average monthly temperature and rainfall were the same across the entire country, although Afghanistan is geographically diverse [48]. Change in temperature does not necessarily equate to a rise in malaria in some parts of the country, particularly in regions which experience high temperatures on average; in fact, higher temperature (>31 C^0^) can have an inhibitory effect on the mosquito life cycle [49]. Thus, the negative correlation of temperature in some corners of the country is perhaps balanced by a positive correlation in others. Thus, vegetation seems to be a better predictor of malaria at the country level, because greenness is not only an indicator for bountifulness of environments for growth of mosquitos, but also moisture and appropriate temperature, both of which are relevant to malaria. A study of malaria patterns in different Afghan provinces, using local scale data from 2004 to 2007, also pointed to vegetation as the strongest predictor of malaria [50], as well as another geospatial study of vivax malaria, the dominant type in the country in 2005 [9].

Declines in malaria incidence in Afghanistan and elsewhere have prompted a paradigm shift from the national level action to region-limited interventions, especially in malaria hotspots. Indeed, since the early 2000s, Afghanistan has steadily come closer to realizing such a scenario. However, these efforts have recently been hampered for two reasons: (1) The required funds to initiate the next phase of the malaria control strategy have yet to be realized, despite efforts to shift the strategy to more local control efforts since 2012 (personal communication with an official in the Ministry of Public Health). (2) The recent deterioration of security (particularly since 2014) throughout the country has raised concerns about potential increases in malaria incidence [51]. The government’s lack of effective territorial control over many malaria burdened areas make it untenable to move toward region-focused initiatives. In light of Afghanistan’s current context, it is tenable that a national-level predictive tool is still very much required, particularly one that can be cost-efficient, to at least ensure the success in the first phase of malaria control in this resource-poor setting.

Most malaria studies in Afghanistan have either focused upon general trends of infection in recent years [45], or the implementation of preventive measures and their effects on the burden of malaria [44]. In general, studies which have assessed the correlation of environmental variables and malaria incidence have tended to be focused on smaller geographic scales [52, 53]. Analysis conducted in this paper complements these efforts by attempting to build a predictive tool that can be used to forecast malaria cases at a national level based on observations from a passive surveillance system that is currently in place. In a country such as Afghanistan, where infrastructure is limited, a system that can accurately predict future malaria trends would be a great asset for public health planning and resource allocation. In addition, proposed model forecasts malaria incidence based solely on passive surveillance data and widely available climate indices, enabling short-term predictions that may provide useful indicators of lapses in malaria control in a setting of ongoing civil unrest. Not only were proposed models able to forecast malaria up to one year ahead with minimum data inputs, but they also provide a means to better understand malaria dynamics in a setting disproportionately affected by lack of resources, ongoing civil unrests, and climate change [54].

## Additional files

**Additional file 1.** Malaria Metadata.

**Additional file 2.** Area-Averaged of CMG 0.05 Deg Monthly EVI monthly 0.05 ().

**Additional file 3.** Area-Averaged of Air temperature at surface (Daytime/Ascending) ().

**Additional file 4.** Area-Averaged of Precipitation Rate monthly 0.25 ().

**Additional file 5.** Area-Averaged of Relative Humidity at Surface (Daytime/Ascending) ().

**Additional file 6: Annex 1.** Right side: Autocorrelation (ACF) and partial autocorrelation (PACF) functions of the residuals from ARIMA model (1, 0, 1) × (1, 0, 1)_12_ on log-transformed, differenced data. Left side: ACF and PACF of the residuals from ARIMA model (4, 0, 1) × (1, 0, 1)_12_ on log-transformed, differenced data.

**Additional file 7: Annex 2.** Pairwise correlation between malaria ARIMA model residuals and external regressor residuals at different lags, after pre-whitening (removing trends and seasonality and fitting ARIMA models to each) (first table). In preliminary analyses, statistically significant correlation was observed between rain and humidity (r = 0.7032, p < 0.001); subsequently, humidity was dropped after it was found not to add meaningful information. Had we not performed pre-whitening, statistically significant correlations existed between malaria and other variables at every lag we analyzed.

**Additional file 8: Annex 3.** Approximate estimation of malaria suspects expected up to December 2016, based on Model 2 with 2-Lag Vegetation. This estimate may be taken with following considerations: 1- Assuming linear trend of malaria stays the same as the Model predict. 2- Incidences not reported to the system remain small or negligible. The numbers calculated are incidence rate per 10 000 of service users in the country
