# Supplementary material for: Time series analysis of malaria in Afghanistan: using ARIMA models to predict future trends in incidence
Source: Malar J. 2016 Nov 22;15:566. doi: 10.1186/s12936-016-1602-1 (PMC5120433; doi:10.1186/s12936-016-1602-1)
Supplement: Supplementary file 6 — Additional file 6: Annex 1. Right side: Autocorrelation (ACF) and partial autocorrelation (PACF) functions of the residuals from ARIMA model (1, 0, 1) × (1, 0, 1)12 on log-transformed, differenced data. Left side: ACF and PACF of the residuals from ARIMA model (4, 0, 1) × (1, 0, 1)12 on log-transformed, differenced data. [file 12936_2016_1602_MOESM6_ESM.docx]

**Annex 1.**


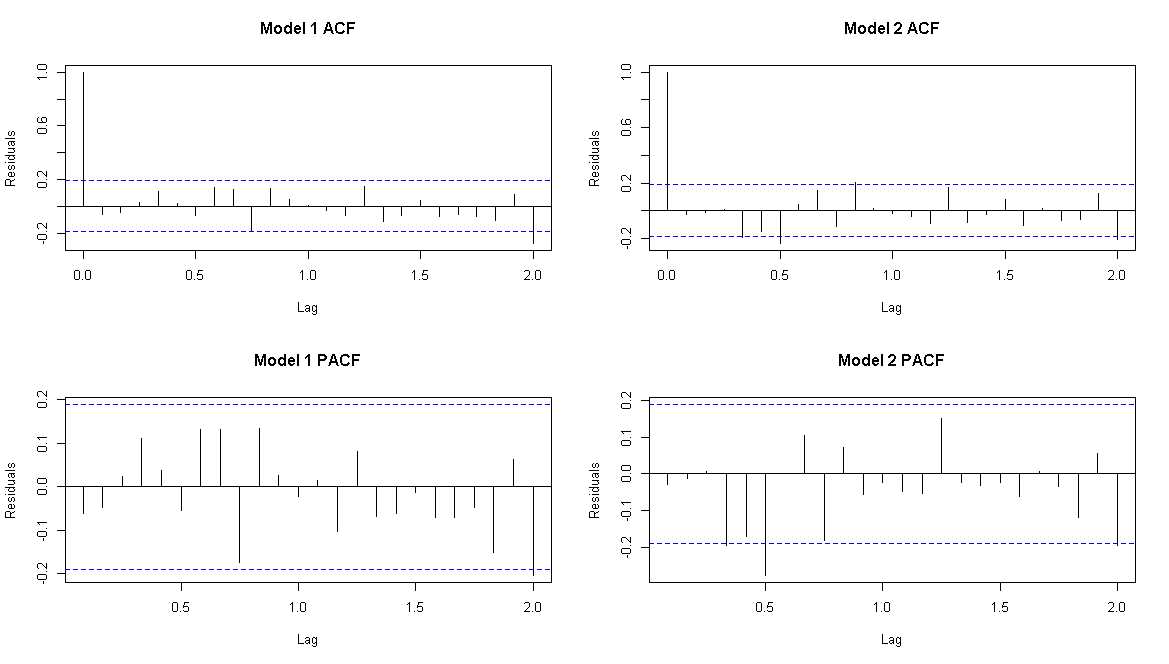


Right side: Autocorrelation (ACF) and partial autocorrelation (PACF) functions of the residuals from ARIMA model (1, 0, 1) × (1, 0, 1)_12_ on log-transformed, differenced data. Left side: ACF and PACF of the residuals from ARIMA model (4, 0, 1) × (1, 0, 1)_12_ on log-transformed, differenced data
