# Supplementary material for: Time series analysis of malaria in Afghanistan: using ARIMA models to predict future trends in incidence
Source: Malar J. 2016 Nov 22;15:566. doi: 10.1186/s12936-016-1602-1 (PMC5120433; doi:10.1186/s12936-016-1602-1)
Supplement: Supplementary file 7 — Additional file 7: Annex 2. Pairwise correlation between malaria ARIMA model residuals and external regressor residuals at different lags, after pre-whitening (removing trends and seasonality and fitting ARIMA models to each) (first table). In preliminary analyses, statistically significant correlation was observed between rain and humidity (r = 0.7032, p < 0.001); subsequently, humidity was dropped after it was found not to add meaningful information. Had we not performed pre-whitening, statistically significant correlations existed between malaria and other variables at every lag we analyzed. [file 12936_2016_1602_MOESM7_ESM.docx]

**Annex 2.**

Pairwise correlation between malaria ARIMA model residuals and external regressor residuals at different lags, after pre-whitening (removing trends and seasonality and fitting ARIMA models to each) (first table). In preliminary analyses, statistically significant correlation was observed between rain and humidity (r = 0.7032, p < 0.001); subsequently, humidity was dropped after it was found not to add meaningful information. Had we not performed pre-whitening, statistically significant correlations existed between malaria and other variables at every lag we analyzed.

After pre-whitening

| Malaria | Vegetation | | Rain | | Temperature | |
| --- | --- | --- | --- | --- | --- | --- |
|  | **Coefficient** | ***p-value*** | **Coefficient** | ***p-value*** | **Coefficient** | ***p-value*** |
| Lag 0 | 0.0256 | 0.7848 | -0.0381 | 0.6847 | 0.1382 | 0.1424 |
| Lag 1 | -0.1435 | 0.1261 | 0.0115 | 0.9028 | 0.0313 | 0.7423 |
| Lag 2 | **0.2012*** | **0.0318** | 0.0326 | 0.7304 | -0.0459 | 0.6310 |
| Lag 3 | -0.0989 | 0.2972 | -0.0611 | 0.5205 | 0.1043 | 0.2760 |

Before pre-whitening

| Malaria | Vegetation | | Rain | | Temperature | |
| --- | --- | --- | --- | --- | --- | --- |
|  | **Coefficient** | ***p-value*** | **Coefficient** | ***p-value*** | **Coefficient** | ***p-value*** |
| Lag 0 | 0.3244 | <0.001 | -0.4583 | <0.001 | 0.4965 | <0.001 |
| Lag 1 | -0.3173 | <0.001 | -0.4554 | <0.001 | 0.4668 | <0.001 |
| Lag 2 | 0.3085* | <0.001 | -0.4503 | <0.001 | 0.4635 | <0.001 |
| Lag 3 | -0.3027 | <0.001 | -0.4559 | <0.001 | 0.4625 | <0.001 |
