# Supplementary material for: Time series analysis of malaria in Afghanistan: using ARIMA models to predict future trends in incidence
Source: Malar J. 2016 Nov 22;15:566. doi: 10.1186/s12936-016-1602-1 (PMC5120433; doi:10.1186/s12936-016-1602-1)
Supplement: Supplementary file 8 — Additional file 8: Annex 3. Approximate estimation of malaria suspects expected up to December 2016, based on Model 2 with 2-Lag Vegetation. This estimate may be taken with following considerations: 1- Assuming linear trend of malaria stays the same as the Model predict. 2- Incidences not reported to the system remain small or negligible. The numbers calculated are incidence rate per 10 000 of service users in the country [file 12936_2016_1602_MOESM8_ESM.docx]

**Annex 3.**

Approximate estimation of malaria suspects expected up to December 2016, based on Model 2 with 2-Lag Vegetation. This estimate may be taken with following considerations:

1- Assuming linear trend of malaria stays the same as the Model predict.

2- Incidences not reported to the system remain small or negligible.

The numbers calculated are incidence rate per 10000 of service users in the country.

| **Month** | **Mean** | **95% confidence interval** | |
| --- | --- | --- | --- |
|  |  | Lower Bound | Upper Bound |
| Oct 2015 | 77.70705 | 60.61113 | 99.62504 |
| Nov 2015 | 98.50192 | 59.5395 | 162.9612 |
| Dec 2015 | 78.71841 | 44.80328 | 138.3066 |
| Jan 2016 | 43.20793 | 23.40606 | 79.76245 |
| Feb 2016 | 27.83427 | 14.3444 | 54.01039 |
| Mar 2016 | 24.96286 | 12.32505 | 50.55915 |
| Apr 2016 | 21.87296 | 10.34047 | 46.26737 |
| May 2016 | 29.97978 | 13.6388 | 65.89925 |
| Jun 2016 | 46.60464 | 20.39277 | 106.5079 |
| July 2016 | 67.24388 | 28.40707 | 159.1766 |
| Aug 2016 | 79.50628 | 32.41472 | 195.0117 |
| Sep 2016 | 88.56821 | 34.94947 | 224.4477 |
| Oct 2016 | 104.0376 | 39.7255 | 272.4651 |
| Nov 2016 | 108.2879 | 38.86536 | 301.715 |
| Dec 2016 | 87.62357 | 29.53227 | 259.983 |
